# Effect of Hemolysis Regarding the Characterization and Prognostic Relevance of Neuron Specific Enolase (NSE) after Cardiopulmonary Resuscitation with Extracorporeal Circulation (eCPR)

**DOI:** 10.3390/jcm12083015

**Published:** 2023-04-21

**Authors:** Franz Haertel, Josephine Babst, Christiane Bruening, Jurgen Bogoviku, Sylvia Otto, Michael Fritzenwanger, Thomas Gecks, Henning Ebelt, Sven Moebius-Winkler, P. Christian Schulze, Ruediger Pfeifer

**Affiliations:** 1 Department of Cardiology and Intensive Care, University Hospital Jena, Am Klinikum 1, 07747 Jena, Germany; 2 Department of Cardiology and Intensive Care, University Hospital Halle (Saale), Ernst-Grube-Str. 40, 06120 Halle (Saale), Germany; 3 Department for Internal Medicine II, Katholisches Krankenhaus “St. Johann Nepomuk”, Haarbergstr. 72, 99097 Erfurt, Germany

**Keywords:** neuron specific enolase, hemolysis, hypoxic brain injury, ECMO, CPR, Cerebral Performance Category Scale, intensive care

## Abstract

Background: Hemolysis, a common adverse event associated with veno-arterial extracorporeal membrane oxygenation (VA-ECMO), may affect neuron-specific enolase (NSE) levels and potentially confound its prognostic value in predicting neurological outcomes in resuscitated patients without return of spontaneous circulation (ROSC) that require extracorporeal cardiopulmonary resuscitation (eCPR). Therefore, a better understanding of the relationship between hemolysis and NSE levels could help to improve the accuracy of NSE as a prognostic marker in this patient population. Methods: We retrospectively analyzed the records of patients who received a VA-ECMO for eCPR between 2004 and 2021 and were treated in the medical intensive care unit (ICU) of the University Hospital Jena. The outcome was measured clinically by using the Cerebral Performance Category Scale (CPC) four weeks after eCPR. The serum concentration of NSE (baseline until 96 h) was analyzed by enzyme-linked immunosorbent assay (ELISA). To evaluate the ability of individual NSE measurements to discriminate, receiver operating characteristic (ROC) curves were calculated. Serum-free hemoglobin (fHb, baseline until 96 h) served as a marker for identifying a confounding effect of parallel hemolysis. Results: 190 patients were included in our study. A total of 86.8% died within 4 weeks after ICU admission or remained unconscious (CPC 3–5), and 13.2% survived with a residual mild to moderate neurological deficit (CPC 1–2). Starting 24h after CPR, NSE was significantly lower and continued to decrease in patients with CPC 1–2 compared to the group with an unfavorable outcome of CPC 3–5. In addition, when evaluating on the basis of receiver operating characteristic curves (ROC), relevant and stable area under the curve (AUC) values for NSE could be calculated (48 h: 0.85 // 72 h: 0.84 // 96 h: 0.80; *p* < 0.01), and on the basis of a binary logistic regression model, relevant odds ratios for the NSE values were found even after adjusting for fHb regarding the prediction of an unfavorable outcome of CPC 3–5. The respective adjusted AUCs of the combined predictive probabilities were significant (48 h: 0.79 // 72 h: 0.76 // 96 h: 0.72; *p* ≤ 0.05). Conclusions: Our study confirms NSE as a reliable prognostic marker for poor neurological outcomes in resuscitated patients receiving VA-ECMO therapy. Furthermore, our results demonstrate that potential hemolysis during VA-ECMO does not significantly impact NSE’s prognostic value. These findings are crucial for clinical decision making and prognostic assessment in this patient population.

## 1. Introduction

The most established biomarker for assessing the severity of hypoxic brain injury (HBI) after resuscitation is the neuron-specific enolase (NSE) [1]. NSE is detectable as an enzyme of glucose metabolism in the cytoplasm of neurons of the central and peripheral nervous system and in neuroendocrine tissue, with a biological half-life of approx. 20 h [2,3,4]. Damage to the structures mentioned (e.g., through ischemic processes) leads to the release of the enzyme into the extracellular space and to an increase in the serum concentration [2,3,4].

The neurological assessment of resuscitated patients who receive veno-arterial extracorporeal membrane oxygenation (VA-ECMO) can be difficult, especially during the first days after cardiopulmonary resuscitation (CPR), since patients often have to be sedated, which limits the information of clinical examination as well as from diagnostic test such as electroencephalography (EEG).

During VA-ECMO support, the release of NSE to the plasma can be attributed to some degree to hemolysis due to blood–tubing/rotor contact [5]. Therefore, an artificial elevation of the levels of NSE does not necessarily indicate an injury of the central nervous system, and NSE in this context should be viewed critically as a prognostic parameter [5,6]. Despite some studies suggesting that NSE is a non-specific biomarker, various other studies have shown positive results [1,5,7,8,9,10,11,12,13,14].

However, the identification and characterization of new or existing biomarkers in the process of early evaluation are crucial for predicting poor neurological outcomes, such as death or permanent loss of consciousness, in order to adapt therapeutic measures on the basis of an existing or the alleged living will. On the other hand, resuscitated patients with unimproving myocardial pump function are, in certain cases, prospective candidates for heart transplantation or a permanent cardiac assist device, respectively. This may illustrate the importance of an early and reliable assessment of the individual neurological long-term prognosis and the essential role of NSE serum levels.

Recent studies involving VA-ECMO patients have demonstrated the utility of serial NSE level measurements in assessing neurological outcomes [7,15]. However, these studies also point to the potential confounding effect of underlying hemolysis that makes NSE interpretation difficult.

### Aim of This Study

The aim of this study is to investigate the prognostic role of NSE and the impact of potential hemolysis in resuscitated patients during VA-ECMO support to determine the neurological outcome.

## 2. Methods

### 2.1. Patients

This retrospective data collection analyzes selected parameters from patients who were admitted to the cardiac intensive care unit (ICU) at the University Hospital Jena between 2004 and 2021 and who were treated with VA-ECMO as extracorporeal CPR (eCPR) because no return of spontaneous circulation (ROSC) was achieved during conventional CPR within at least 20 min in the context of out-of- or in-hospital resuscitation (OHCA/IHCA). Patients who received a VA-ECMO without prior resuscitation were excluded. Patients who qualified were treated with therapeutic hypothermia to a target temperature of 33 ± 0.5 °C (degrees Celsius) for approx. 24 h.

### 2.2. Primary Endpoint

The primary endpoint of this study is the neurological status of the resuscitated patients four weeks after eCPR, represented by the Cerebral Performance Category Scale (CPC).

The CPC was determined four weeks after CPR to assess the neurological outcome of resuscitated patients. This grading system has been used in previous studies [16]. In this study, the group labeled ‘unfavorable outcome’ (UO) included all patients who either died within four weeks after eCPR (CPC 5), remained comatose (CPC 4), or had a severe neurological impairment (CPC 3). All resuscitated patients who regained consciousness within four weeks are summarized in the “favorable outcome” (FO) group. The extent of the neurological deficit varies inside this group from moderate (CPC 2) to mild disabilities (CPC 1).

### 2.3. VA-ECMO

All patients were provided with ECMO support from the manufacturer Maquet© (Rastatt, Germany) using a preassembled, standard tubing set (PLS–system, Maquet©, Rastatt, Germany). This included a Rotaflow RF-32 centrifugal pump (Maquet©, Rastatt, Germany), a QADROX^®^ oxygenator (Maquet©, Rastatt, Germany), and standard tubing, which were operated via the Rotaflow console (Maquet©, Rastatt, Germany). The circuit was established through complete lower body cannulation of femoral vessels using sheath sizes 17–21 F (arterial) and 19–25 F (venous). In addition, all patients received antegrade limb perfusion via an extra 7 F bypass cannula (CruraSave^®^ Femoral-Perfusion Set, free life medical GmbH©, Aachen, Germany).

Criteria for ECMO implantation at our facility include CPR lasting more than 20 min without ROSC, regardless of IHCA or OHCA, age between 18 and 80 years, cardiac arrest with immediate bystander CPR, and anticipated uninterrupted resuscitation from collapse until ECMO implantation, whether performed manually or using mechanical chest compression devices. Ultimately, the attending interventional cardiologist makes an individual clinical decision based on these criteria.

### 2.4. Neuron-Specific Enolase and Serum-Free Hemoglobin

Serum samples were obtained at baseline, 24 ± 2, 48 ± 2, 72 ± 2, and 96 ± 2 h after the arrest and assessed for NSE levels [ng/mL; 1 ng/mL = 1 µg/L] as this is part of the standard laboratory workup for resuscitated patients. An enzyme-linked immunosorbent assay (ELISA) was performed using the LIAISON XL^®^ (DiaSorin©, Dietzenbach, Germany; reference cut off: 12.5 ng/mL). Serum-free hemoglobin (fHb, [µmol/L]) analyses were performed photometrically from a lithium heparin sample using the c502 modul^®^ of the Cobas 8000^®^ (Roche©, Basel, Switzerland, reference cut off: 12.4 µmol/L).

### 2.5. Patient Data

The data for this study were gathered from the electronic medical records of the university hospital using two patient data management systems, COPRA^®^ (COPRA System GmbH, Berlin, Germany) and SAP^®^ (Walldorf, Germany). To ensure anonymity, all data were anonymized.

### 2.6. Statistical Analyses

Statistical data analysis was performed using SPSS^®^ Statistics (version 26.0, SPSS Inc., IBM, Armonk, NY, USA). Normal distribution was analyzed by Kolmogorov–Smirnov test. Differences in frequency of nominally scaled parameters were compared by means of Pearson’s chi-squared test. Metric variables are expressed as mean ± standard deviation, and tests on differences were performed by Student’s t-test for independent and dependent variables. To quantify the predictive value of NSE, receiver operating characteristic (ROC) curves were generated, and their respective area under the curve (AUC) values were described. Adjusted AUCs were used to express combined predictive probabilities from binary logistic regression models. Cut-off values were calculated using Youden’s index derived from ROC curves analysis for NSE, for which the patients differ in CPC outcome (CPC 3–5 vs. CPC 1–2). Cut-off values were determined using the maximum value for Youden’s index. The basis for the test decision was a significance level of *p* < 0.05.

## 3. Results

### 3.1. Patients and eCPR Associated Data

Between January 2004 and December 2021, 190 patients were included in this study. All patients were mechanically ventilated during VA-ECMO insertion, unresponsive to verbal commands, sedated, and received inotropic and/or vasopressor support. Within four weeks after the cardiac arrest, 86.8% (n = 165) died, remained unconscious, or presented severe neurological impairment and constituted the “unfavorable outcome” group (UO; CPC 3–5). A total of 13.2% (n = 25) of the patients were allocated to the “favorable outcome” group (FO; CPC 1–2). Selected baseline patient characteristics, resuscitation-associated data, and data on VA-ECMO therapy of the two groups are summarized and compared in Table 1.

**Figure 1:**
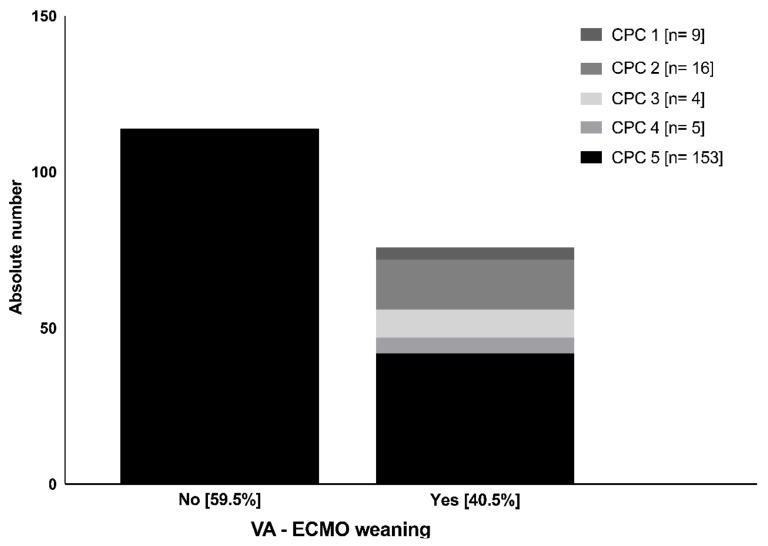
Number of cases of each CPC scale group after four weeks regarding VA-ECMO weaning status. CPC = cerebral performance category scale; VA-ECMO = veno-arterial extracorporeal membrane oxygenation.

Weaning from the VA-ECMO was not successful in 113 patients (59.5%) who subsequently died under ongoing circulatory support (CPC 5, Figure 1). Weaning from VA-ECMO was successful in 77 patients (40.5%; CPC 1–4, Figure 1). In 56 of these patients (72.7%), the circulatory support could be terminated, and VA-ECMO could be removed under stable cardiopulmonary conditions without the need for further circulatory support. In contrast, further mechanical circulatory support (MCS) was necessary for five patients (6.5%) via left ventricular assist device (LVAD), for two patients (2.6%) via Impella, and seven patients (9.1%) via intraaortic balloon pump (IABP). One patient received a heart transplant (1.3%). However, 43 of the 77 patients weaned from VA-ECMO (55.8%) died within four weeks after CPR. Only 25 of the 190 patients (13.2%) survived longer with a moderate to good neurological outcome (CPC 1–2), and 5 patients (2.6%) remained in a persistent vegetative state of unresponsiveness (CPC 4).

### 3.2. Wake-Up Attempt and Neurological Status

**Figure 2:**
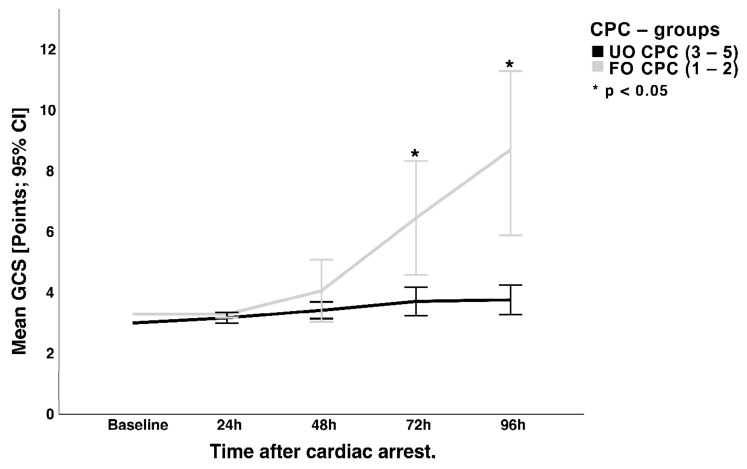
Mean GCS levels of the two CPC groups during a wake-up attempt regarding the first 96 h after VA-ECMO initiation. GCS = Glasgow coma scale; CPC = cerebral performance category scale; UO = unfavorable outcome (CPC 3–5); FU = favorable outcome (CPC 1–2); CI = confidence interval.

For 134 of the 190 patients (70.5%), sedation was interrupted 48–96 h after eCPR in a wake-up attempt to reduce the impact on consciousness (so-called “diagnostic window”). Patients in the CPC 1–2 group showed steady and continuing neurological improvements after 48 h as a reflection of improving consciousness measured with the Glasgow coma scale (GCS, Figure 2).

### 3.3. NSE Serum Concentration and Hemolysis

**Figure 3:**
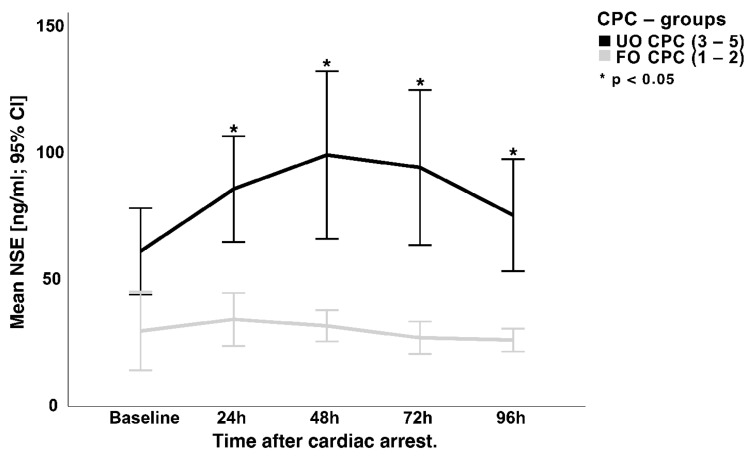
Mean NSE levels compared between the two CPC outcome groups. CPC = cerebral performance category scale; NSE = neuron-specific enolase; *p* = level of significance; CI = confidence interval.

Figure 3 illustrates the course of the NSE serum concentrations up to 96 h after cardiac arrest. Regarding differences between the two outcome groups, significantly lower and decreasing NSE serum concentrations were found in patients with a favorable outcome (CPC 1–2) after 24 h (24 h: 33.5 ± 22.4 ng/mL; 48 h: 27.5 ± 12.8 ng/mL; 72 h: 25.9 ± 15.1 ng/mL; 96 h: 24.2 ± 9.3 ng/m). In contrast, the levels of NSE serum concentration in patients with an unfavorable outcome continue to rise, reaching a peak after 48–72 h (24 h: 79.1 ± 57.6 ng/mL; 48 h: 98.4 ± 87.5 ng/mL, 72 h: 87.5 ± 79.7 ng/mL; 96 h: 68.7 ± 52.1 ng/mL; *p* < 0.01).

**Figure 4:**
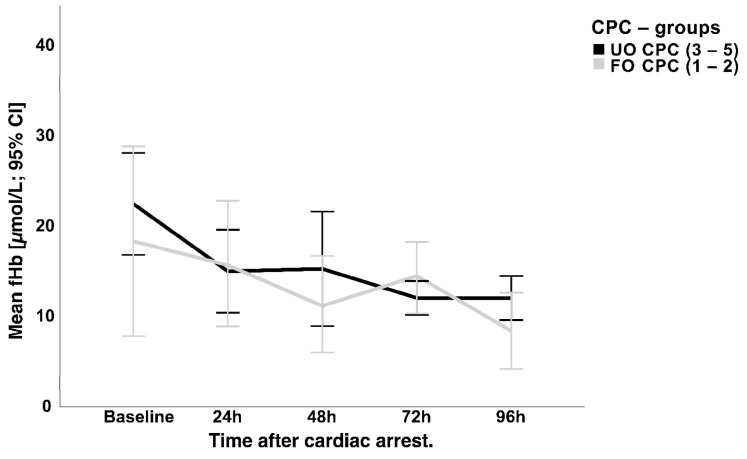
FHb levels compared between the two CPC outcome groups during the first 96 h after VA-ECMO initiation. CPC = cerebral performance category scale; fHb = free hemoglobin; CI = confidence interval.

Regarding the quantification of hemolysis, serum-free hemoglobin (fHb) measurements were initially high in the total study population at baseline (22.8 ± 19.7 µmol/L) but decreased rapidly and remained uneventful during the observation period (24 h: 17.2 ± 13.8; 48h: 16.5 ± 14.3; 72 h: 16.1 ± 12.3; 96 h: 15.9 ± 12.4 µmol/L) without a significant difference between the two outcome groups (Figure 4). No correlation between NSE and fHb could be found on the respective days (24 h: r = 0.14; *p* = 0.21; 48 h: r = 0.13; *p* = 0.28; 72 h: r = 0.12; *p* = 0.25; 96 h: r = 0.097; *p* = 0.47).

**Figure 5:**
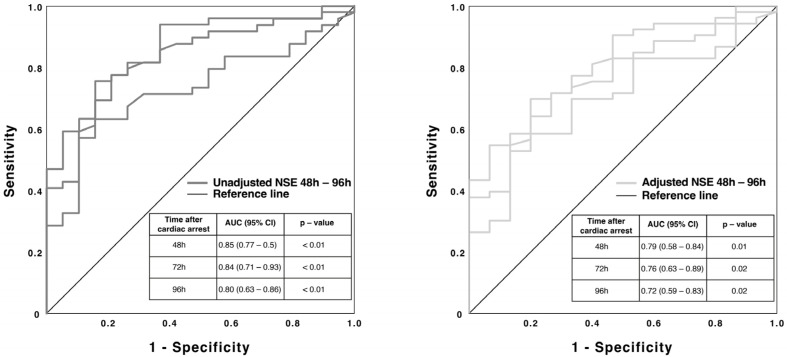
ROC curves of unadjusted NSE (**left** image) and adjusted NSE (**right** image) regarding the prediction of the two outcome groups starting 48 h after VA-ECMO initiation. ROC = receiver operating characteristics; NSE = neuron-specific enolase; AUC = area under the curve; CI = confidence interval; *p* = level of significance.

Figure 5 contains the results of the unadjusted ROC-curve analysis for the NSE serum concentration after 48 h. The calculated significant AUC-values of 0.8 and greater offer a significance level to predict neurological outcomes according to the two groups (CPC 1–2 and CPC 3–5) four weeks after eCPR.

A binary logistic regression model was used to calculate AUCs of the combined predictive probabilities adjusted for hemolysis (fHb). These AUCs remained significant (Figure 5). A mean cut-off value for the NSE after 48 h using Youden’s index could be approximated to 55.9 pg/mL (normal < 55.9 pg/mL > pathological) with a specificity of 100% and a sensitivity of 59%.

## 4. Discussion

Most previous clinical studies evaluating prognostic markers in resuscitated individuals excluded patients treated with VA-ECMO, since ROSC has always been a prerequisite for inclusion. The determination of the prognosis is clinically essential, not only because of the decision whether the intensive therapy should be continued, but also whether the patient is suitable for further therapy intensification, such as LVAD implantation or heart transplantation. The evaluation of NSE to predict the neurological outcome four weeks after resuscitation in patients under the terms of VA-ECMO treatment was the main focus of this work and will be discussed below. 

### 4.1. NSE and Neurological Outcome

Patients with a favorable neurological prognosis showed significantly lower serum NSE concentrations as early as the second day after eCPR, while an increase in the NSE serum concentration was recorded in patients with an unfavorable outcome up to 48 h after eCPR. In the ROC curve analyses, a high ability to discriminate between favorable and unfavorable neurological outcomes was recorded for the NSE serum concentration on day 2, 3, and 4 after eCPR, respectively.

These results confirm that serum concentration of NSE can be seen as a reliable marker for prognostication of unfavorable neurological outcomes under the use of VA-ECMO as well. The distinct and significant differences in NSE serum levels between the outcome groups and their time course are comparable to patient cohorts with [15] and without using VA-ECMO [17].

There is currently no uniformly accepted and validated cut-off for the NSE serum concentration for resuscitated persons undergoing conventional CPR that would allow a reliable assessment of the neurological prognosis [18], especially as different treatment strategies might influence the prognostic yield of NSE [19,20]. Observing the time course of the NSE serum concentration over a period of the first three to five days after eCPR seems to allow an assessment of the prognosis, as stated by the study by Schrage et al. [15]. Patients who exhibited a consistent decline in NSE levels demonstrated a significant tendency towards a favorable neurological prognosis in comparison to patients with progressively rising NSE levels [15].

In the current literature, there are investigations regarding the prognostic potential of NSE serum concentration in patients on VA-ECMO therapy in various contexts.

Reuter et al. [10] demonstrated in a prospective study involving 103 patients that elevated NSE values 48–72 h after VA-ECMO initiation were associated with relevantly increased 28-day mortality and poor functional outcome (=modified Rankin scale (mRS) of 4–6, clinically corresponding to severe disability or death). Although reported in medians, the work characterized a decreasing course of the NSE values during the first 24–72 h (37 ng/mL (26–51 ng/mL) to 25 ng/mL (19–37 ng/mL), which were much lower compared to the NSE findings of the total population of the presented study [10]. However, these results should be put under special consideration and cannot simply be related to the results of our study, as the cited work analyzed patients in refractory cardiogenic shock from a heterogenous collective (VA-ECMO was initiated in 41 surgical patients (40%) and in 62 medical patients (60%)) [10]. Furthermore, only 26 patients (25%) received pre-ECMO CPR [10], as opposed to 100% in our study population.

Floerchinger et al. [21] investigated the association between the peak levels of serum NSE and possible neurological injuries detected by cerebral imaging in 159 patients. As a major finding, the authors showed that particularly severe diffuse cerebral injury was relevantly associated with the highest NSE peaks (208.5 ± 126.0 ng/mL) [21]. NSE serum peak levels in patients without any lesion (78.8 ± 50.1 ng/mL) were comparable with those having focal ischemic lesions (70.1 ± 30.2 ng/mL) [21], which is a finding that relates to the content of our results [21]. Patients discharged from the hospital had significantly lower NSE peaks than patients who deceased after resuscitation (74.9 ± 56.9 ng/mL vs. 138.9 ± 108.4 ng/mL; *p* < 0.0001) [21]. Regarding the prediction of neuronal injury using NSE, receiver operating curve analysis for NSE serum peaks around 48 h after VA-ECMO implantation showed an AUC of 0.733 (*p* < 0.0001, 99% CI 0.62–0.85) [21], comparable to our adjusted AUC results. However, this work also involved a heterogeneous patient collective, as approximately 37% had no imminent pre-ECMO resuscitation but received the VA-ECMO due to low-cardiac output and shock [21].

The study by Schrage et al. [15] aimed to evaluate the predictive value of Neuron-specific enolase (NSE) in VA–ECMO patients. NSE was measured in post-CPR ECMO patients, and neurologic status was evaluated according to CPC [15]. Results showed that NSE can be used to assess the neurologic outcome in post-CPR patients on ECMO [15]. The best discrimination for poor neurological outcome was seen with NSE measurements after 48 h, and specificity was highest if using serial NSE measurements at all three time points [15]. These results are consistent with our findings and suggest that NSE is a reliable predictor of neurological outcomes in this patient population.

### 4.2. NSE and Hemolysis

The prognostic value of the serum concentration of NSE must be critically assessed in patients undergoing ECMO therapy, since an increase in the biomarker can be caused not only by damage to neuronal structures due to hypoxia, but also by mechanical damage to erythrocytes and thrombocytes by external circulatory support systems [5,6]. Therefore, in the present study, the serum concentration of free hemoglobin (fHb) was used as a marker of hemolysis.

The mean serum values of the fHb of all patients within the first five days of treatment were marginally elevated and are, therefore, signs of moderate hemolysis. In the period under consideration, no significant differences in the fHb serum concentrations could be detected between the two outcome groups, so it can be assumed that the level of the NSE serum concentration was not significantly affected by hemolysis caused by the VA-ECMO.

Although the aforementioned study by Reuter et al. [10] presented positive findings regarding the predictive value of NSE, potential confounders of elevated NSE serum levels, such as hemolysis, were not considered in detail. A brief statement was provided in the methodology section, stating that samples exhibiting visible hemolysis were excluded [10].

The study by Floerchinger et al. [21] also measured free hemoglobin levels 24 h after VA-ECMO. Their findings, with an average of 149 ± 162 mg/L [21], were consistent with the results of our study. However, in contrast to their findings, our study revealed no significant correlation between higher NSE levels and increased levels of free serum hemoglobin.

In a recent observational retrospective registry study conducted by Ben-Hamouda et al. [7], comatose patients after cardiac arrest were evaluated, including 397 patients without ECMO and 50 patients undergoing ECMO. This study found differences in serum NSE levels between the two groups (NSE levels were higher in patients with ECMO, *p* < 0.001) [7], which may be related to a longer time to return of circulation in patients with ECMO. However, the authors highlight that it is difficult to determine whether the higher NSE values of patients receiving ECMO are solely due to more severe brain damage or hemolysis [7]. Therefore, they recommend a combination of at least two “poor outcome” criteria for neurological prognosis [7].

A study by Schrage et al. [15] confirmed that NSE can be used to assess the neurologic outcome in patients after CPR on ECMO. The serial NSE measurements suggest that ECMO therapy leads to an “NSE noise” that increases with NSE levels due to mild, underlying hemolysis [15]. Nevertheless, this does not mask the significant release of NSE from the brain’s white matter in severe brain injury cases [15]. It is worth mentioning in this context that the authors point out the accuracy of the presented NSE cut-off values for predicting poor neurologic outcomes as they have been verified in an external cohort, which further reinforces the credibility of their results and limits the influence of hemolysis [15].

Although performed in a different patient collective of 97 patients under mechanical circulatory support by ventricular assist devices (VADs) or total artificial hearts (TAHs), a study by Geisen et al. [22] aimed to investigate the correlation between NSE and indicators of hemolysis. The researchers analyzed NSE, haptoglobin, hemopexin, free hemoglobin, lactate dehydrogenase activity, platelet counts, and total bilirubin in plasma and assessed major cerebral events in 97 patients [22]. The results showed that NSE correlated with markers of hemolysis and was influenced by intravascular hemolysis [22]. Thus, the study suggests caution in using NSE to assess cerebral damage in these patients [22].

### 4.3. Calculation of a New Cut-Off Value

The cut-off value of the NSE calculated for the present patient cohort, which indicates a poor neurological outcome, is almost congruent with the cut-off values described in the literature for patients after CPR without VA-ECMO treatment [1,8,23,24]. This may indicate that there are uniform mechanisms in the pathophysiology of HBI following CPR and that NSE from possible hemolysis under VA-ECMO is of no relevance. However, some publications propose higher cut-off values greater than 100 pg/mL [10,21,25]. 

While different cut-off values for NSE can vary significantly, this may be due to various factors, including different testing methods or sampling times, as well as variations in normal values across different laboratories. As a result, the authors suggest that, in addition to using a cut-off value for predicting poor neurological outcomes, it is important to track the NSE serum levels over several days to gain a better understanding of the patient’s prognosis.

### 4.4. Strengths and Limitations of This Study

Although our study has defined inclusion criteria for eCPR, it is important to acknowledge that these criteria and the therapy standards have evolved and been modified over a period of 13 years in response to new research findings and changing protocols and procedures. This evolution may be considered a potential limitation of our study, as it introduces the possibility of confounding factors that could affect the results.

However, a wide range of therapy data, laboratory parameters, and neurological diagnostic findings was recorded and analyzed from a typical patient population in a maximum-care hospital, which allows a comprehensive assessment of patients undergoing VA-ECMO therapy and enables conclusions to be drawn about the outcome of the resuscitated patient.

The validity of the present results is limited, in particular, by the retrospective single-center study design. The survival rate and the neurological outcome of the examined patients after eCPR are worse compared to current studies of the last few years, whereby the comparability of the data is difficult due to different patient populations.

## 5. Conclusions

Patients with unfavorable neurological outcomes after eCPR show significantly higher NSE levels during the first 96 h than patients with good outcomes. In this selected patient cohort, hemolysis does not seem to have an influence on the group differences. NSE serum levels >55.9 ng/mL 48 h after CPR seem to be indicative of an unfavorable neurological outcome.

## Figures and Tables

**Table 1:** Baseline characteristics, resuscitation-associated parameters, and clinical/technical VA-ECMO data of the total study population and the two outcome groups (CPC 1–2 and CPC 3–5).

	Study Population	CPC 1–2	CPC 3–5	*p*-Value *
	**(n = 190)**	**(n = 25)**	**(n = 165)**	
**Biometrics**				
Age (years–mean ± SD)	60.1 ± 15.6	61.2 ± 20.2	60 ± 15.1	0.72
Male–(n (%))	141 (74)	16 (64)	125 (75.8)	0.42
Female–(n (%))	49 (26%)	9 (36)	40 (24.2)	0.42
BMI (kg/m^2^-mean ± SD)	28.5 ± 6.1	27.2 ± 7.2	28.6 ± 5.9	0.46
Number of comorbidities (mean ± SD)	2.2 ± 1.1	2.3 ± 1.5	2.2 ± 1.1	0.89
Left ventricular ejection fraction (%–mean ± Std)	25.1 ± 15.5	35.3 ± 15.3	24.7 ± 15.4	0.002
**CPR data**				
Duration of CPR (minutes–mean ± SD)	53.5 ± 38.1	31.9 ± 24.4	55.2 ± 38.6	0.004
Primary rhythm				
VT/VF (n (%))	92 (48)	15 (60)	77 (46.7)	0.06
PEA/Asystole (n (%))	98 (52)	10 (40)	88 (53.3)	0.54
Cumulative dose of adrenaline (mg-mean ± SD)	5.4 ± 5.2	2.5 ± 2.4	5.5 ± 5.4	0.007
Number of defibrillations (n (%))	3.6 ± 8.4	1.9 ± 4.3	3.6 ± 8.6	0.33
TTM (n (%))	148 (77.9)	20 (80)	128 (77.6)	0.34
Bystander CPR (n (%))	91 (47.8)	11 (44)	80 (48)	0.25
In-hospital CPR (n (%))	133 (70)	17 (68)	116 (70.3)	0.58
**Major etiology of CPR**				
STEMI (n (%))	83 (43.6)	13 (52)	70 (42.4)	0.56
**VA-ECMO data**				
Intervall collapse-implantation [(minutes–mean ± SD)]	52.4 ± 42.6	32.6 ± 31.9	67.3 ± 43.3	<0.001
Pulsatile flow after ECMO implantation (n (%))	144 (76.3)	25 (100)	119 (72.1)	<0.001
Duration of VA-ECMO support (hours ± SD)	103.4 ± 112.1	121.9 ± 131.9	103.2 ± 108.3	0.44
Initial liters/minute (mean ± SD)	4.2 ± 0.9	3.8 ± 1.1	4.3 ± 0.9	0.013
Initial pump flow index (mL/minute/kg-mean ± SD)	51.02 ± 14.4	48.8 ± 17.6	51.3 ± 14.1	0.43

BMI = body mass index, CPC = cerebral performance category, CPR = cardiopulmonary resuscitation, N = absolute number, PEA= pulseless electrical activity, SD = standard deviation, STEMI = ST-elevation myocardial infarction, TTM = therapeutic temperature management, VA-ECMO = veno-arterial extracorporeal membrane oxygenation, VF = ventricular fibrillation, and VT = ventricular tachycardia, * CPC 1–2 vs. CPC 3–5.

## Data Availability

The data presented in this study are not publicly available due to local legal restrictions on data safety.
